# 2381 Childhood adversity, attachment style, and home visiting engagement

**DOI:** 10.1017/cts.2018.240

**Published:** 2018-11-21

**Authors:** Kelly M. Bower, Deborah Gross, Phyllis Sharps

**Affiliations:** Johns Hopkins University School of Medicine

## Abstract

OBJECTIVES/SPECIFIC AIMS: This case-control study aims to determine the relationships among childhood adversity, attachment style, and the likelihood of accepting or declining a referral for HV. The study will serve as a pilot to inform the power analysis of a subsequently proposed full-scale study. METHODS/STUDY POPULATION: Using a case-control study design, 25 women who decline HV referral (cases) will be compared with 25 women who accept HV referral (controls) on their exposure to childhood adversity and attachment style. Women who are eligible for the study are English-speaking mothers who have been offered HV services by Health Care Access Maryland. Surveys are administered in-person, either in the participant’s home or at another location (e.g., public library), based on participant preference. The dependent variable is participant’s verbal response to the HV referral (accept/decline). The independent variable, childhood adversity, will be measured using the Philadelphia Urban Adverse Childhood Experiences (ACEs) Survey and the Attachment Style Questionnaire (ASQ). Control variables include demographics (i.e., age, race, education, employment, housing, marital status), obstetric history (i.e., previous preterm birth, miscarriage, fetal death, infant death, abortion), and current psychosocial risk factors (i.e., history of substance use, intimate partner violence, depression). Descriptive comparisons will be done for the independent and control variables in controls versus cases. Bivariate analysis will examine associations between the odds of being a case and ACE score and ASQ score. Multivariate logistic regression models will be used to examine the relationship between ACE total and ASQ score; exposure to ACE in cases versus controls; and the odds of an avoidant and anxious attachment styles in cases versus controls. RESULTS/ANTICIPATED RESULTS: We hypothesize that (a) higher ACE scores will be positively associated with a higher level of avoidant attachment; (b) higher ACE scores will be positively associated with declining a HV referral; and (c) higher levels of avoidant attachment will be associated with declining a HV referral. DISCUSSION/SIGNIFICANCE OF IMPACT: Racial inequities in birth outcomes are pervasive and unjust. Non-Hispanic Black women experience births that result in infant mortality, fetal mortality, preterm birth, and low birth weight babies at more than double the rate of non-Hispanic White women in Baltimore and nationally. Prenatal and early childhood home visiting programs have been found to decrease maternal smoking and hypertensive disorder which are associated with PTB, reduce closely spaced births which is associated with fetal and infant death, and improve women’s long-term economic self-sufficiency, child health and social outcomes. However, as community-based programs, these services are not reaching the majority of eligible women in low-income urban settings—women who are also disproportionately burdened with poor pregnancy-related health outcomes. Considering the potential to improve outcomes, the importance of eliminating health disparities, and the national and local investment in HV services, it is vital to understand why some women are not enrolling in prenatal HV programs. The findings from this and subsequent studies will inform the translation of evidence-based HV program outreach efforts for women with complex social history. It will inform the design of enhanced outreach and engagement efforts of HV programs to more reliably engage women.

